# Processing of Composite Electrodes of Carbon Nanotube Fabrics and Inorganic Matrices via Rapid Joule Heating

**DOI:** 10.1021/acsami.2c17901

**Published:** 2023-01-17

**Authors:** Shegufta Upama, Anastasiia Mikhalchan, Luis Arévalo, Moumita Rana, Afshin Pendashteh, Micah J. Green, Juan J. Vilatela

**Affiliations:** † Department of Materials Science & Engineering, Texas A&M University, College Station, Texas77843, United States; ‡ IMDEA Materials Institute, Getafe, Madrid28906, Spain; § Institut für Anorganische und Analytische Chemie, University of Münster, Münster48149, Germany; ∥ Artie McFerrin Department of Chemical Engineering, Texas A&M University, College Station, Texas77843, United States

**Keywords:** carbon nanotube fabric, MoS_2_, Joule heating, direct current, nanostructured network, composite, tensile properties, electrical conductivity

## Abstract

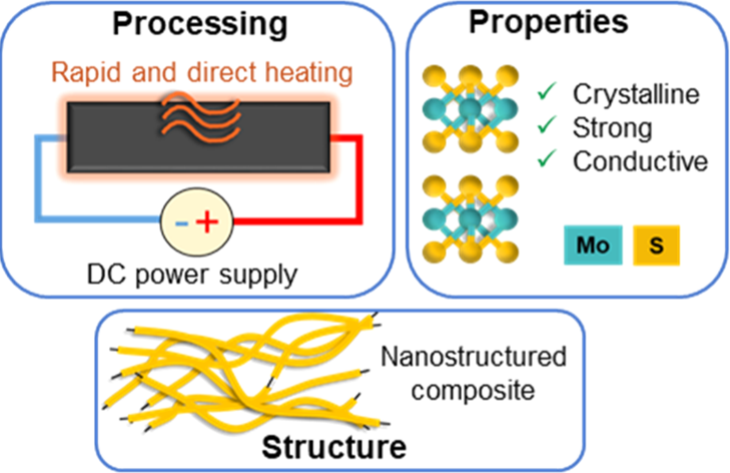

Composites of nanocarbon network structures are interesting materials, combining mechanical properties and electrical conductivity superior to those of granular systems. Hence, they are envisaged to have applications as electrodes for energy storage and transfer. Here, we show a new processing route using Joule heating for a nanostructured network composite of carbon nanotube (CNT) fabrics and an inorganic phase (namely, MoS_2_), and then study the resulting structure and properties. To this end, first, a unidirectional fabric of conductive CNT bundles is electrochemically coated with MoS_2_. Afterward, the conformally coated inorganic phase is crystallized via heat generated by direct current passing through the CNT ensemble. The Joule heating process is rapid (maximum heating rate up to 31.7 °C/s), enables accurate temperature control, and takes only a few minutes. The resulting composite material combines a high electrical conductivity of up to 1.72 (±0.25) × 10^5^ S/m, tensile modulus as high as 8.82 ± 5.5 GPa/SG, and an axial tensile strength up to 200 ± 58 MPa/SG. Both electrical and mechanical properties are orders of magnitude above those of wet-processed nanocomposites of similar composition. The extraordinary longitudinal properties stem from the network of interconnected and highly aligned CNT bundles. Conductivity and modulus follow approximately a rule of mixtures, similar to a continuous fiber composite, whereas strength scales almost quadratically with the mass fraction of the inorganic phase due to the inorganic constraining realignment of CNTs upon stretching. This processing route is applicable to a wide range of nanocarbon-based composites with inorganic phases, leading to composites with specific strength above steel and electrical conductivity beyond the threshold for electronic limitations in battery electrodes.

## 1 Introduction

Carbon nanotubes (CNTs) are nanoscale building blocks that possess an extraordinary combination of properties—electrical and thermal conductivities comparable to those of copper,^1^ theoretical tensile modulus and strength exceeding those of carbon fiber,^2,3^ and ultrahigh surface area and aspect ratio—that make them ideal as reinforcement in composites. Assembling CNTs into macroscopic aligned fibers and arrays (e.g., yarns, tows, and fabrics) allows for these axial properties to be utilized on the macroscale, opening diverse applications such as electrodes for energy storage,^4−9^ structural reinforcement,^10^ sensors,^11^ actuators,^12^ and drug delivery.^13^

CNTs are among a range of conductive materials (e.g., carbon fiber, carbon black, graphene, and MXenes) that heat up directly and rapidly in response to electric fields. These electric fields can be produced using alternating or direct current (also called Joule heating), or frequencies in the microwave or radio-frequency range (known as dielectric heating).^14^ When in a matrix, these conductive nanomaterials can heat up a composite from within. These methods provide a targeted, controlled, and out-of-oven processing technique with several applications in material synthesis and manufacturing—synthesizing 2D materials^15^ and high-temperature carbides,^16^ curing composites without molds,^17−19^ recycling carbon fiber from spent composites,^20^ bonding multimaterial surfaces together,^21^ and pyrolyzing waste into graphene.^22^

Our group has previously shown that a preformed network of CNT fabrics (CNTFs) can be used as the scaffold for growing inorganic phases (such as LFP, ZnO, MnO_2_, TiO_2_, MoS_2_) to form a nanostructured network composite.^4−9^ These electrochemically active materials bind firmly to the CNT network and give flexible and mechanically robust structures with large surface area. Moreover, since the CNT network is preserved after growing the inorganic phase, there is low charge-transfer resistance across the interface.

Such morphology is particularly relevant in battery electrodes, where integrating two nanomaterials—a current collector and an electrochemically active material—into a single structure not only maximizes the stress/charge transfer between the building blocks but also eliminates the need for polymeric binders, additives, or metallic current collectors while enhancing battery performance.^23,24^ The absence of a metallic foil current collector enables the fabrication of complex-shaped devices such as multifunctional structural batteries for vehicles^25^ and thin, flexible, and stretchable batteries.^9,26^

In our prior work, we showed that integrating an inorganic phase (namely, MoS_2_) into a preformed network of CNTF gives a flexible hybrid electrode. When used as an anode for lithium-ion batteries, our composite material outperforms most high-capacity structural electrodes and provides high in-plane and out-of-plane electrical conductivity (10^3^ and 1.2 S/m, respectively).^4^ Beyond lithium-ion battery chemistry, 2H-phase MoS_2_ is particularly appealing due to its layered structure and large interlayer spacing (∼6.15 Å), providing a suitable host for reversible intercalation of bulky ions such as sodium or potassium.^27,28^ However, a major hurdle in the development of these battery chemistries is that the aqueous electrolytes lead to the corrosion of the metallic current collectors.^29^ This challenge can be overcome using nanocarbon current collectors.

These composite electrodes can provide electrical and mechanical properties beyond those obtained through conventional processing routes. In this quest, it is imperative to establish clearer relationships between their structure and bulk anisotropic properties.

In this work, we used electrochemical deposition, followed by Joule heating, for the controlled fabrication of CNTF/inorganic nanocomposites. Accordingly, an in-house setup was developed to carry out Joule heating in a controlled fashion, and later study the effect of processing on the structure and mechanical properties. It is demonstrated that Joule heating provides a direct means to inside-out crystallization of the conformal MoS_2_ layer, while enabling excellent interfacial interaction between the MoS_2_ and the underlying CNT surface. The significant impact of the proposed procedure in processing CNTF/inorganic composites, in terms of radically reduced processing time (and potentially energy) as well as improved mechanical properties of the composite, was demonstrated compared to conventional methods (e.g., wet-processed and furnace-heated). The obtained data support the improved time and energy efficiency of the new processing technique as well as high mechanical and electrical properties of the final composite. The new method produces an electrode morphology superior to that obtained with conventional slurry-based processing methods with respect to the tensile properties (modulus and strength up to 8.82 ± 5.5 GPa/SG and 200 ± 58 Mpa/SG, respectively) and axial electrical conductivity [up to 1.72 (±0.25) × 10^5^ S/m]. Although this composite based on MoS_2_-coated CNTF is employed as a model system, it is noted that the concept can be extended to other inorganic/CNT composite systems, hence opening new opportunities for high-performance composites with combined properties.

## 2 Experimental Section

### 2.1 Materials

Toluene, ferrocene, thiophene, ammonium tetrathiomolybdate (99.9%), sodium sulfate (99.99%), and aluminum (99%) were used. All chemicals were purchased from Sigma and used without further purification.

### 2.2 Methods

#### 2.2.1 Synthesis of Directly Spun CNTFs

The CNTFs were synthesized using gas-phase chemical vapor deposition (CVD). The precursors for carbon, the catalyst, and the promoter were toluene, ferrocene, and thiophene, respectively. The precursors were introduced from the top of a vertical furnace at 1300 °C under a hydrogen atmosphere. The synthesis conditions were chosen so as to produce bundles of predominantly few-layer CNTs, a significant fraction of which is collapsed due to their large diameter.^30^ The CNTs were directly drawn as CNT bundles from the bottom of the furnace onto a rotating drum. Successive layers of CNT bundles were collected for 20 min to form a nonwoven, unidirectional CNTF.

#### 2.2.2 Synthesis of CNTF/MoS_2_ Composite

The CNTF/MoS_2_ composite was fabricated in two steps.

(i) The pristine CNTF was functionalized using a gas-phase ozone treatment (Jelight UVO-cleaner model 18, U.S.) for 20 min (e.g., 10 min each side). Such treatment renders the fabric hydrophilic, ensuring wetting of the mat during the following step—i.e., electrodeposition of the inorganic phase in an aqueous deposition bath.
(ii) MoS_2_ was deposited onto the functionalized CNTF using electrochemical deposition in a three-electrode setup. The working, reference, and counter electrodes were the as-prepared functionalized CNTF, a Ag/AgCl (3 M) electrode, and a Pt mesh, respectively. The electrolyte solution consisted of 10 mM ammonium tetrathiomolybdate and 0.1 M sodium sulfate in deionized water. A constant potential of −1.8 V was applied for different durations between 1 and 30 min to deposit various mass fractions of MoS_2_. Afterward, the samples were thoroughly washed in water and ethanol and then dried under ambient conditions.

#### 2.2.3 Processing of CNTF/MoS_2_ Composite

The direct current (DC) heating setup was inside a vacuum chamber (Pfeiffer Vacuum high vacuum chamber, DN 300) that was maintained under a vacuum/argon atmosphere (∼3.10 mbar). Initially, the chamber was evacuated, and later, an Ar flow was continuously fed to provide an inert atmosphere and prevent any unwanted oxidation of the composite. The CNTF/MoS_2_ samples were cut using sharp scissors into rectangular strips (0.3 × 2.0 cm) and soldered to electrical wires along the middle of either end of the fabric. Then, the samples were connected in series to a DC power supply (Delta Elektronika SM660-AR-11) and a 100 Ω resistor, which was added as a safety precaution to limit the current flowing through the sample and prevent sample damage. Two digital multimeters (Keysight 34465A) were used to measure the voltages of the resistor (*V*_resistor_) and the DC power supply (*V*_source_). A LabView program was used to plot and analyze the voltage and current of the sample according to the following equations
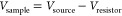
1
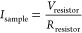
2

The DC voltage was manually modulated at a rate of ∼50 °C/min until the sample reached the target temperature (i.e., 450 °C), and the temperature was maintained between 5 and 15 min. Ramping up the DC voltage led to an instant rise in temperature, which was monitored using an infrared thermometer (Optris CTlaser pyrometer).

The conventionally annealed sample was heated using the same conditions in our prior work^4^ at 600 °C in a horizontal furnace under a continuous flow of argon with a heating ramp of 5 °C/min.

#### 2.2.4 Characterization

The morphology and crystalline phase of the samples were characterized using field-emission scanning electron microscopy (SEM) (FEI Helios NanoLab 600i), Raman spectroscopy (Renishaw, fitted with a 532 nm laser source), and powder X-ray diffraction (PXRD, Cu Kα radiation, Empyrean, PANalytical Instruments). To check for the uniformity of MoS_2_ coating through the thickness of the sample, we used an adhesive tape (Sellotape) to peel off successive layers of the sample and then performed Raman spectroscopy on each of the exfoliated layers.

The composite mass fraction was measured using thermogravimetric analysis (TGA Q50, TA Instruments) in air using a sequential temperature program. First, the temperature was raised from room temperature to 100 °C with a ramp rate of 10 °C/min and a dwell time of 20 min, to ensure the removal of physically adsorbed moisture. Then, the temperature was ramped at 10 °C/min up to 1000 °C with no dwell time. The mass fraction for the composite at 673 °C was subtracted from that for the functionalized fabric at the same temperature (see Figures S1 and S2). This value was taken as the MoO_3_ mass fraction, assuming that all the MoS_2_ oxidized to MoO_3_ in a 1:1 molar ratio. Finally, the mass fraction of MoS_2_ was calculated from the MoO_3_ mass fraction.

The longitudinal electrical resistance was measured using the four-probe technique to avoid a contact contribution to resistance. The resistivity (ρ) and electrical conductivity (σ) were calculated from the resistance (*R*), length (*L*), and cross-sectional area (*A*) of the sample as follows
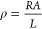
3
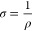
4

The out-of-plane electrical resistance was measured using a two-probe technique.

Uniaxial tensile tests were performed in the force-controlled mode using a dynamic mechanical analyzer machine (DMA 850, TA Instruments) equipped with an 18 N load cell and tensile clamps designed for uniaxial deformation. The cyclic tensile tests were conducted for 10 cycles up to a maximum of 1% strain rate using a Favimat textile testing machine (Textechno, Germany). Both the CNTF/MoS_2_ and CNTF specimens were cut into rectangular strips having a width of 3 mm and an initial gauge length of 15 mm. The load–displacement curves were recorded, and the specific values of tensile strength, elastic modulus, and elongation-to-break were calculated afterward. The mass of each sample was measured using a high-precision microbalance. The lateral dimensions of the samples were measured using a ruler, and the thickness was determined using a high-precision micrometer. The density of each sample was calculated by dividing the mass by the volume. Specific properties, determined from knowledge of the load and specific gravity, were used to eliminate uncertainty with determination of cross sections and enable direct comparison of tensile properties for other CNTF-based samples with different porosities.

## 3 Results and Discussion

**Figure 1:**
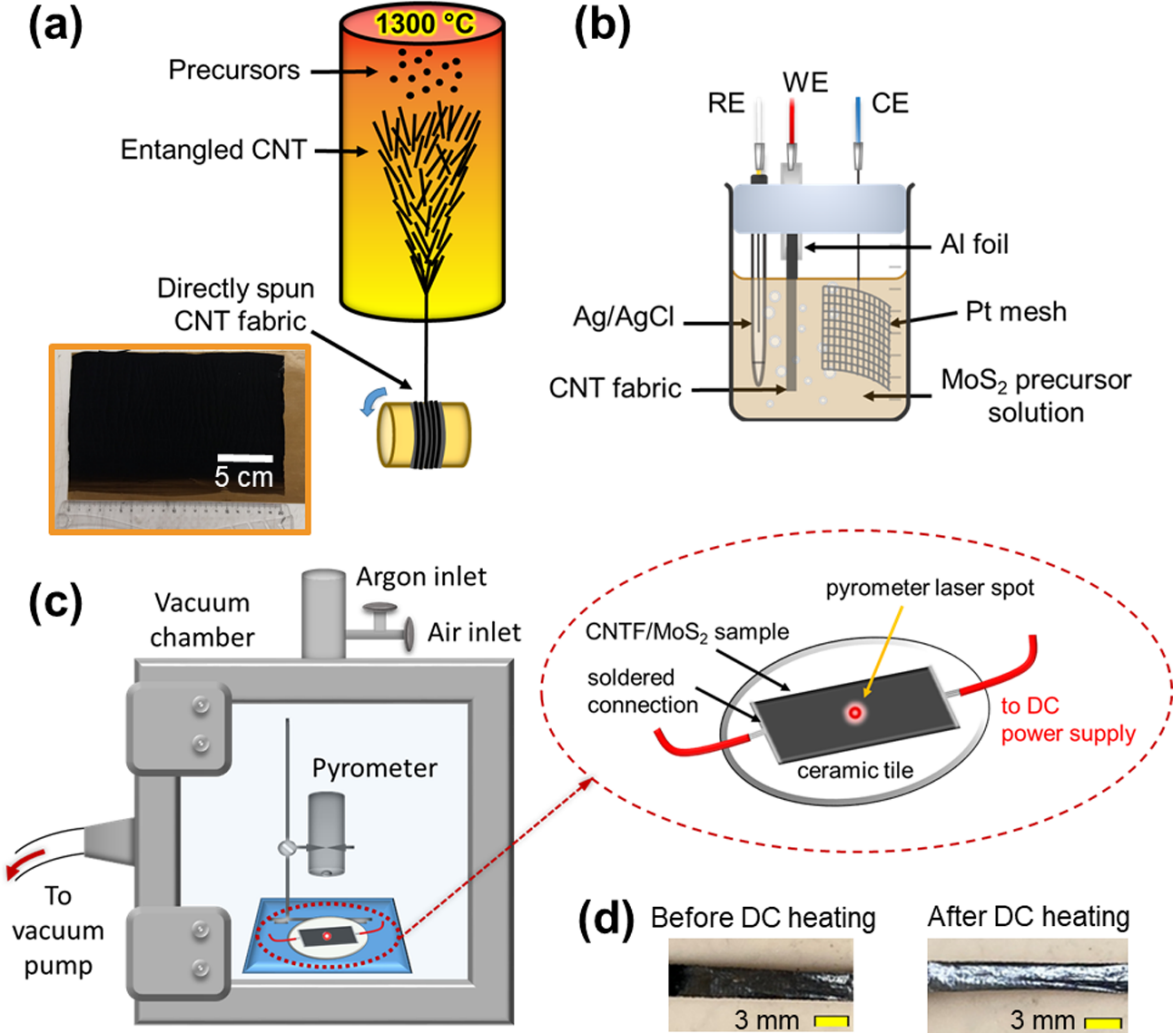
Schematic of the techniques used to fabricate CNTF/MoS_2_ hybrids: (a) synthesis of CNTF using floating catalyst CVD (FC-CVD). The inset shows a digital image of a CNTF. (b) Electrodeposition of MoS_2_ in a three-electrode setup. (c) DC heating setup with a zoomed-in view of the sample, showing electrical connections to DC power supply. (d) Digital images of a CNTF/MoS_2_ (60% MoS_2_) sample before and after DC heating.

The CNTF/MoS_2_ composites were fabricated in four steps. First, the CNTF was synthesized directly from the gas phase using floating catalyst CVD by utilizing the van der Waals forces between the nanotubes (Figure 1a). The CNT aerogel was collected onto a rotating drum for 20 min to form a nonwoven, free-standing CNTF. Second, the CNTF was ozone-treated to introduce oxygen-containing functional groups.^31^ The functionalization serves two purposes, namely, to make the CNTF hydrophilic for processing with an aqueous electrolyte, and to provide nucleation sites for the inorganic phase during the electrodeposition. Third, the functionalized CNTF was coated with MoS_2_, the inorganic phase, using electrochemical deposition (Figure 1b). Finally, the composites were processed using Joule heating to crystallize the inorganic phase (Figure 1c).

The synthesized CNTF is composed mainly of multiwall CNTs (2–4 walls) with a small proportion of single-wall CNTs. It also comprises a small amount of amorphous carbon attached to the surface of CNTs (8–12% wt) and iron catalyst nanoparticles (8–10% wt). The length of the nanotubes is in the micron range, and the crystallinity degree is high (D/G peak intensity ratio ≈ 0.14).^30^ The functionalization protocol using ozone increases the D/G ratio to ∼0.40. Based on previous work, this would roughly correspond to a polarity (i.e., the polar component of CNT surface energy) of 26% and a high surface energy of ∼22 mJ/m^2^.^32^ Transmission electron microscopy (TEM) analysis of the CNTF after functionalization shows the presence of defects such as holes and broken layers owing to the formation of sp^3^ bonds in the newly formed functional groups.^31^ These functional groups are responsible for inducing wetting and swift infiltration of the electrolyte used for electrochemical deposition.

The deposition results in a conformal amorphous MoS_2_ coating, which according to high-resolution TEM is predominantly aligned parallel to the CNTs.^4^ This coating can be crystallized using a heat treatment. Applying DC voltage through the CNTF/MoS_2_ composite generates current within the electrically conductive CNT network in the macroscopic fabric. This generates heat within the CNTF, which in turn transfers heat to the surrounding MoS_2_ phase. As soon as the target temperature (450 °C) is reached (after ∼480 s), the sample turns from dull gray to shiny silver, indicating that MoS_2_ has undergone a phase transition (Figure 1d). While the phase change is instantaneous at temperatures ≥450 °C, we maintained the temperature for ∼10 min to ensure uniform heating of the entire sample and homogeneous crystallization of the inorganic phase.

**Figure 2:**
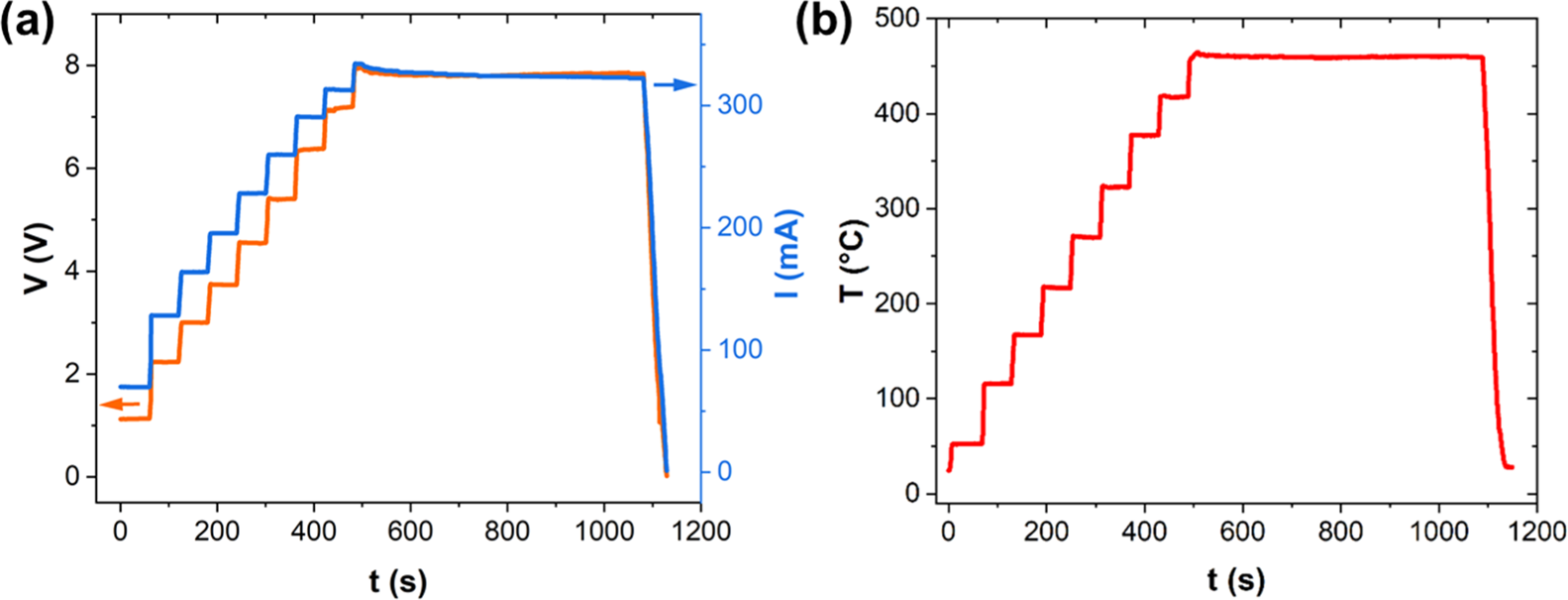
DC heating data for a CNTF/MoS_2_ composite with dimensions 0.3 × 2.0 cm and mass ∼0.80 mg: (a) voltage and current vs time. The DC voltage was ramped in stages until the target temperature (450 °C) was reached. The voltage was then modulated to maintain the temperature at 450 °C for 10 min. (b) Resulting temperature profile with time.

Based on the thermal profile, we achieved high control over the DC heating process (Figure 2). The DC power was controlled by ramping the voltage, which in turn controlled the temperature of the sample. The maximum heating rate was up to 31.7 °C/s (see Movie S1 and Figure S3). The processing method was controllable for all MoS_2_ mass fractions produced. Generally, as the mass fraction of MoS_2_ increases, the power density (i.e., power normalized by the sample mass) decreases (Figure S4).

Unlike conventional heating mechanisms such as conduction and convection, where the heat is transferred from the exterior (i.e., the heat source) to the interior (e.g., the sample), Joule heating generates heat directly from the inside out. It is more energy-efficient because of minimal heat losses to the surroundings. Additionally, Joule heating is a simpler and faster technique because it is not limited by a slow ramp rate (e.g., 5 °C/min), which is essential not only to prevent thermal shock of the ceramic/quartz tube in a tubular furnace but also to ensure a uniform temperature of the heating elements and furnace cavity. Thus, while conventional annealing takes at least 1 h just to ramp up to the target temperature, the entire process of Joule heating MoS_2_ can be accomplished in only ∼8 min. This reduced annealing time may prevent unwanted oxidation of the underlying CNT network. The processing of similar composites using Joule heating can be scaled for large-scale fabrication in a roll-to-roll fashion, potentially with electrical leads on the rolls. Similar concepts have been demonstrated for epoxy-carbon fiber towpregs.^33^ Other alternatives would be to use flat electrical contacts that use pressure, as opposed to soldering, or noncontact electromagnetic heating, such as radio-frequency heating.

**Figure 3:**
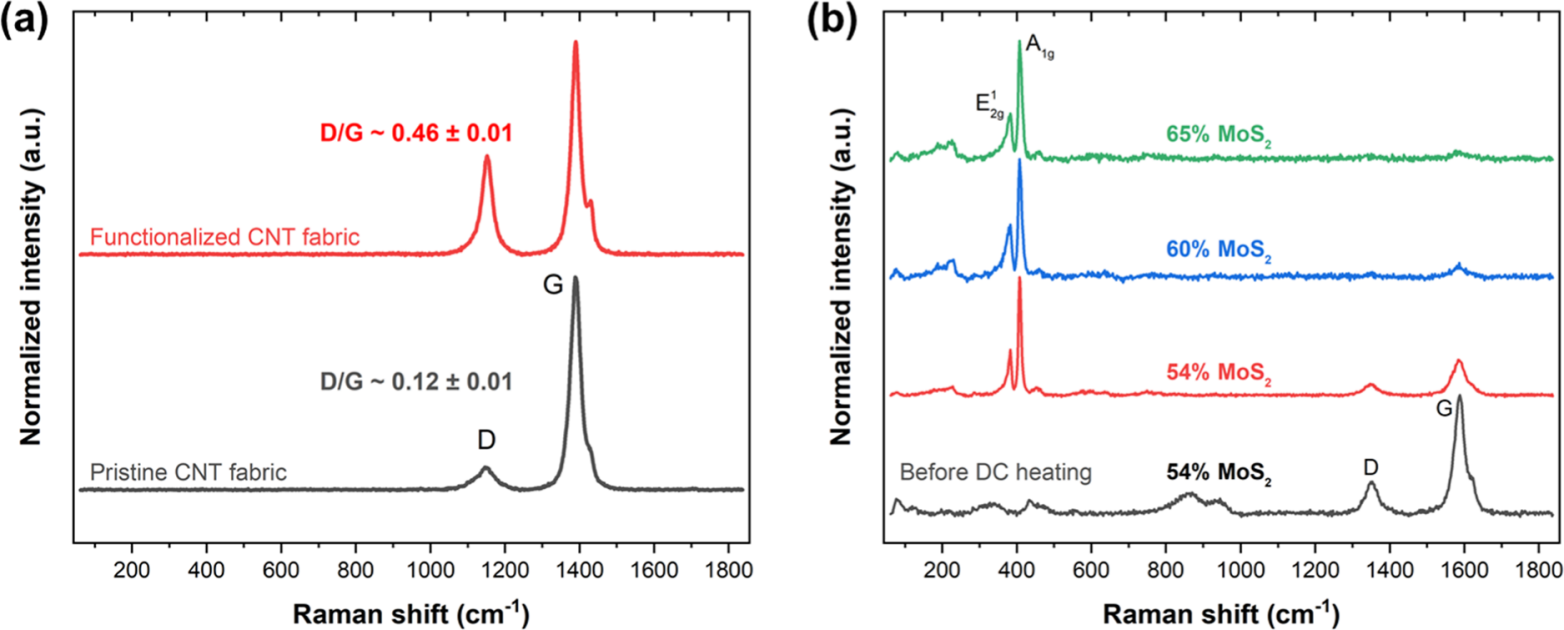
(a) Raman spectra of CNTF before and after functionalization. (b) Raman spectra of CNTF/MoS_2_ composites with different weight fractions before and after DC heating. Here, the MoS_2_ weight fraction was varied from 54 to 65% MoS_2_ by changing the electrodeposition time from 5 to 15 min.

Raman spectroscopy was used to determine the change in surface functionalization due to ozone treatment, as baseline for the composite. The Raman spectra of the pristine CNTF show the symmetric vibrations from defect (D)-rich and graphitic (G) regions of the CNTs at 1348 and 1580 cm^–1^ (Figure 3a). After ozone treatment, which introduces various oxygen-containing functional groups^31^ to the surface of the CNTF, the D and G bands slightly shift to 1349 and 1581 cm^–1^, respectively. After functionalization, the intensity ratio of D/G peaks increases from 0.12 ± 0.01 to 0.46 ± 0.01. Both the higher D/G peak intensity ratio and the G peak shoulder (D′ peak) are associated with defects such as functional groups. Nonetheless, since the D/G peak intensity ratio is less than 1, there are relatively few defects and sp^2^ hybridization is still dominant, which suggests that the electrical and mechanical properties of the CNTs are largely preserved (see Tables 1 and 2).^31,34^

**Table 1:** Morphological Parameters and Electrical Conductivity of CNTF/MoS_2_ Composites Synthesized Using Different Electrodeposition Times

electro-deposition time (min)	MoS_2_ mass fraction (%)	coated CNT bundle diameter (nm)	average MoS_2_ coating thickness (nm)	longitudinal electrical conductivity (S/m)	out-of-plane electrical conductivity (S/m)
0	0	21.1 ± 4.9		2.43 (±0.90) × 10^5^	2.01 (±0.53) × 10^0^
5	54	123 ± 11	51.0	1.72 (±0.25) × 10^5^	1.61 (±0.19) × 10^–1^
7	60	146 ± 19	62.5	8.58 (±1.75) × 10^4^	1.71 (±0.14) × 10^–1^
15	65	160 ± 15	69.5	7.47 (±1.67) × 10^4^	1.23 (±0.20) × 10^–1^
30	68	295 ± 37	137	6.54 (±0.73) × 10^4^	7.45 (±0.93) × 10^–2^

**Table 2:** Mechanical Properties of CNTF/MoS_2_ Composites

MoS_2_ mass fraction (%)	specific modulus (GPa/SG)	specific strength (MPa/SG)	strain to break (%)
0	17.9 ± 1.2	613 ± 48	4.4 ± 0.8
44	8.33 ± 2.7	200 ± 58	3.7 ± 1.1
54	8.53 ± 2.9	131 ± 27	2.0 ± 0.2
60	8.82 ± 5.5	70.7 ± 38	0.91 ± 0.06
65	5.59 ± 0.8	62.3 ± 32	1.4 ± 0.4
68	3.59 ± 0.9	62.1 ± 21	4.0 ± 1.0
100^4,39^	1.00 × 10^–3^	1.00 × 10^–1^	1.7 ± 0.7

The crystalline phase transition of the inorganic phase was examined using Raman spectroscopy and XRD. For the just-deposited CNTF/MoS_2_ composite, we see amorphous peaks for MoS_2_ in addition to CNT signals (Figure 3b). After DC heating the composite, we see characteristic 2H-phase MoS_2_ peaks at ∼382 and ∼408 cm^–1^, which correspond to the in-plane (E_2g_^1^) and out-of-plane (A_1g_) vibrations of the MoS_2_ layers and low-intensity CNT peaks, respectively. The broad peaks around 800–1000 cm^–1^, which can be attributed to amorphous Mo oxide, disappear on DC heating due to volatilization of the Mo oxide species.^35^ For all the mass fractions studied, the intensity ratio of the A_1g_/E_2g_^1^ Raman peaks was ∼2.25, corresponding to the growth of edge-oriented MoS_2_ layers (Figure S5).^36^ The frequency difference between the two peaks was also consistent at ∼25.5 cm^–1^, which corresponds to bulk or multilayer MoS_2_.^37^ The MoS_2_ layers are likely parallel to the CNTs. This can be inferred from the PXRD pattern for the CNTF/MoS_2_ composite (Figure S6), which shows that the basal planes of the 2H crystalline phase of MoS_2_ dominate.

Next, we examine the degree of crystallization and structural quality of the composite using the intensity ratios of the A_1g_/G and D/G peaks, respectively. As the duration of DC heating increases from 5 to 10 min, the A_1g_/G peak intensity ratio decreases from 10.19 ± 0.39 to 5.33 ± 0.18, but the crystallization is more uniform across the sample (Table S1). Further increases in the DC heating duration do not appreciably change the crystallization degree of the sample. The low-intensity peaks at 1346 and 1583 cm^–1^ can be attributed to the CNT bundle core. After crystallization, the intensity ratio of the D/G bands slightly decreases to ∼0.40, which suggests that some of the defects within the functionalized CNTF get healed after Joule heating. As the mass fraction and coating thickness of MoS_2_ increase, more MoS_2_ is excited by the laser beam, so the CNT signals appear less intense relative to the MoS_2_ peaks, demonstrating the enhanced thickness of the deposit.

**Figure 4:**
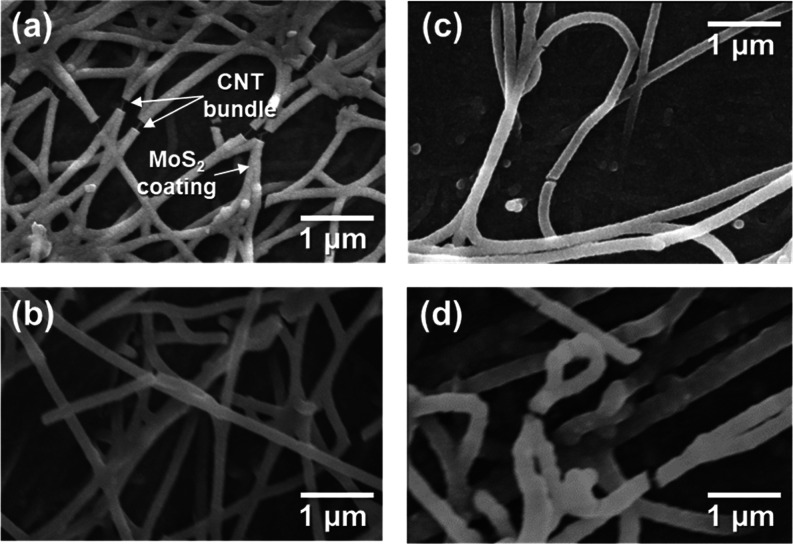
Field-emission-SEM images of CNTF/MoS_2_ composites with different mass fractions (after Joule heating): (a) 54% MoS_2_, (b) 60% MoS_2_, (c) 65% MoS_2_, and (d) 68% MoS_2_. The images show that the conformal coating of MoS_2_ grows in thickness as its mass fraction increases and that the network structure of the composite is preserved through Joule heating.

The composite can be visualized as two connected, continuous, nanostructured phases; the CNTF forms a coated porous network where the pores gradually fill up as MoS_2_ grows conformally around the CNT bundles (Figure S7). After Joule heating, the network of CNT bundles and the morphology of MoS_2_ are preserved, indicating the robustness of the CNTF under this processing technique (Figures 4 and S7). Morphological parameters, such as the diameter of the MoS_2_-coated CNT bundles, the dependence of electrodeposition time (5–30 min) on the MoS_2_ mass fraction, and the electrical conductivity of the composites, are summarized in Table 1 and in Figure S8. [Note that a 44% MoS_2_ composite (i.e., 1 min deposition time) was also used for mechanical characterization in a later section and it was not analyzed further for morphology.] Longitudinal conductivity is close to a rule of mixtures, whereas out-of-plane conductivity drops faster with increasing MoS_2_ fraction. This is expected considering the different envisaged conduction mechanisms, that is, along the CNT network and thus proportional to their fraction, compared to through-thickness, where the MoS_2_ coating between bundles can affect transverse conductivity. Histograms of bundle diameters and MoS_2_ coating determined from SEM are included in Figure S9. The mass fraction has a sublinear dependence on apparent thickness, as expected for a system where the pores fill in, but the supporting bundle network is unaltered, which is the case for the materials in this study (Figure S10).

The CNTF/MoS_2_ core shell becomes thicker as MoS_2_ is deposited around the CNT bundles. The bundle diameter grows from 21.1 nm for the neat CNTF to 123 and 295 nm for the composite as the MoS_2_ deposition time is increased from 5 to 30 min, respectively. In addition, the distance between the E_2g_^1^ and A_1g_ Raman peaks increases from 24.5 to 25.5 cm^–1^ (from 5 to 30 min deposition time) due to the E_2g_^1^ peak shifting to lower frequencies (i.e., redshift) and the A_1g_ peak shifting to higher frequencies (i.e., blueshift). Both of these frequency shifts are associated with changes in molecular packing, such as an increasing number of layers as the MoS_2_ coating grows, resulting in higher interlayer van der Waals forces.^38^

**Figure 5:**
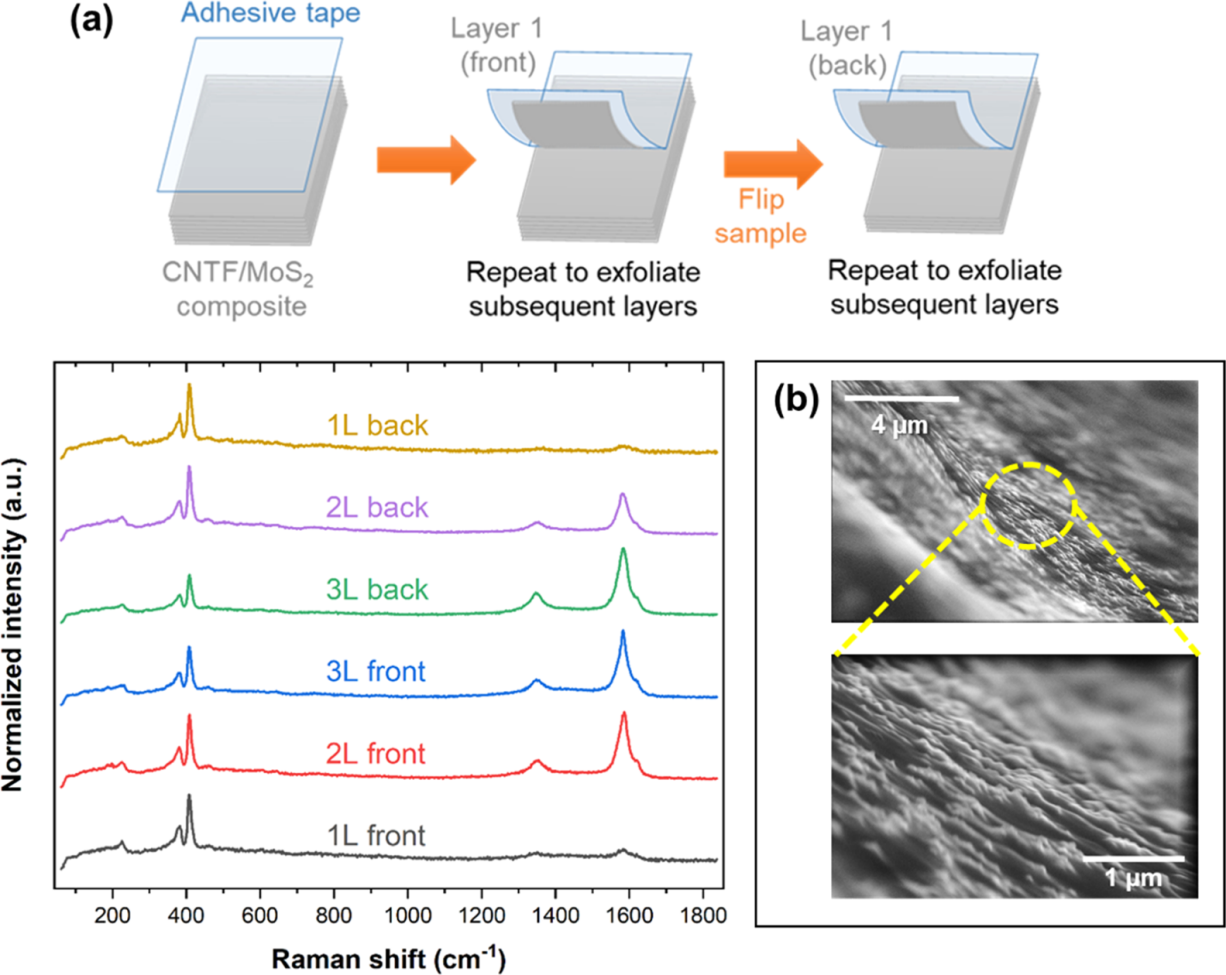
(a) Schematic of the method used to exfoliate layers of CNTF/MoS_2_ (60% MoS_2_) and the corresponding Raman spectra. The uniformity of MoS_2_ coating is seen throughout the thickness of the sample. Here, the top side and opposite side of the sample are labeled as front and back, respectively. (b) SEM images of CNTF/MoS_2_ (60% MoS_2_) along the edge (cross-section) of the sample, showing the continuous MoS_2_ coating (in light gray) layered morphology.

We investigated whether MoS_2_ coats the fabric only superficially or throughout the whole thickness of the samples by analyzing the Raman spectra and SEM morphology at different depths in the composite. Figure 5a shows the methodology for peeling off successive layers of the CNTF/MoS_2_ composite as well as the corresponding Raman spectra. The results indicate that the MoS_2_ coating is uniform throughout the six layers analyzed. As we go from the outer to the inner layers of the composite, crystalline MoS_2_ is seen throughout. The A_1g_/G intensity ratio decreases from 4.35 ± 1.43 to 0.83 ± 0.25, from the outer to the inner layers, respectively. This may be due to excess MoS_2_ deposition on the outer surfaces. SEM images of the sample across the thickness confirm the continuous MoS_2_ coating and show the layered morphology of the composite (Figure 5b).

Finally, we determined the mechanical properties of the CNTF/MoS_2_ material. Figure S11 shows the tensile testing setup, and Table 2 summarizes the results from uniaxial tensile tests. Both the specific modulus and specific strength increase as the CNTF mass fraction increases, indicating that the CNT network bears more load and mechanically reinforces the MoS_2_ matrix. Note that the void fraction was accounted for by normalizing the tensile properties by specific gravity. Compared to our prior work on CNTF/MoS_2_, the specific strength is ∼10 times higher and the specific modulus is 2–3 orders of magnitude higher.^4^ They are also 4 orders of magnitude stronger than bulk MoS_2_ and two above-mentioned high-performance wet-processed composite electrodes with CNTs,^19^ including segregated networks of CNTs with MoS_2_ nanosheets,^39^ NMC microparticles, and Si micro- or nanoparticles.^23^ In fact, on a weight-normalized basis, these composites are stronger than steel.

**Figure 6:**
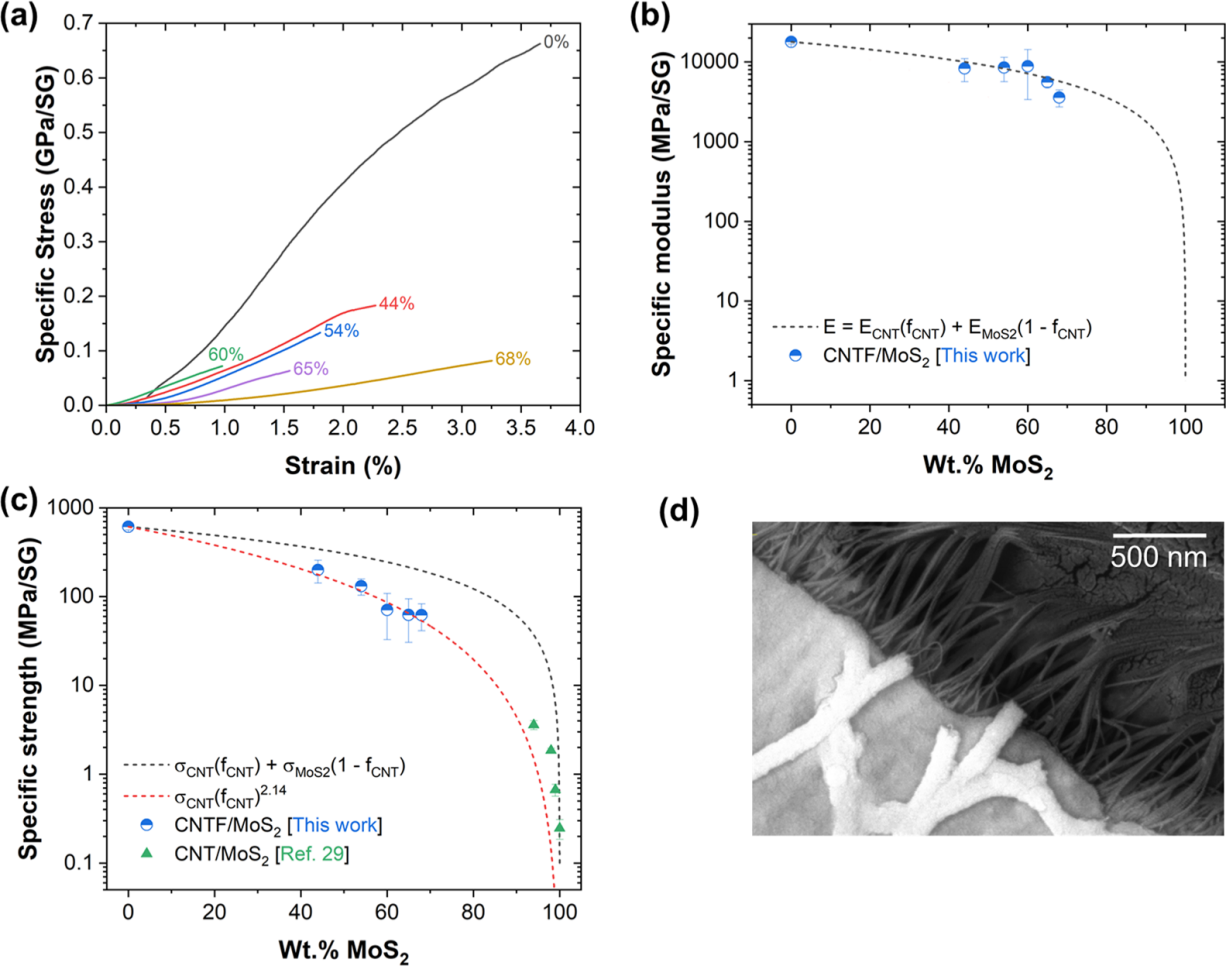
(a) Representative stress–strain plots for CNTF/MoS_2_ specimens with different mass MoS_2_ fractions and functionalized CNTF (i.e., 0% MoS_2_). (b) Comparison of specific tensile modulus with the rule of the mixture model. (c) Comparison of specific tensile strength with the rule of the mixture model, a power law, and data for wet-processed CNT/MoS_2_ composites.^39^ (d) Back-scatter diffraction SEM image of the fracture surface of a 54% MoS_2_ specimen, showing brittle failure and CNT bundle pullout.

Figure 6a shows the representative stress–strain plots, and Figure S12 compares the stress–strain plots for the composite before and after crystallization of the inorganic phase, via either conventional annealing or Joule heating. Although the strain-to-break is identical for the two heating processes, both the longitudinal tensile strength (∼90 MPa/SG) and tensile modulus (∼5 GPa/SG), as well as the longitudinal electrical conductivity [9.93 (±0.79) × 10^4^ S/m] of the conventionally annealed 54% MoS_2_ composite were lower than its Joule-heated counterpart. We hypothesize that the reduced annealing time prevents unwanted oxidation of the CNT network and preserves its mechanical and electrical properties. The presence of the crystalline MoS_2_ coating generally reduces strain-to-break for all mass fractions; nevertheless, significant ductility is preserved. Interestingly, despite a large internal porosity, the material behaves similar to a continuous fiber-reinforced composite. As Figure 6b shows, the longitudinal modulus follows a rule-of-mixture dependence on mass fraction. This indicates that the composite modulus is mainly governed by the modulus of the CNT network, which in turn is controlled by the degree of CNT alignment.^40^ The introduction of MoS_2_ into the fabric is not expected to disrupt the initial degree of CNT alignment; hence, the composite modulus is essentially proportional to the mass fraction of CNTs.

On the other hand, a plot of composite strength for different mass fractions shows a dependence below the rule of mixtures (Figure 6c). Instead, strength follows a power law, with an exponent of 2.14. This is attributed to MoS_2_ filling the pores between bundles and restricting their reorganization under axial deformation of the composite. In the pure fabric, moderate longitudinal deformations produce plastic deformation through realignment and sliding of CNT bundles, increasing the buildup of stress in the fabric network. These mechanisms are impeded in the CNTF/MoS_2_, hence the reduction in strain-to-break and tensile strength despite a proportional modulus. Inspection of fracture surfaces (Figure 6d) clearly shows brittle failure of MoS_2_ and pullout of CNT bundles in the fracture zone. However, the sample with the highest mass fraction shows high strain-to-break. At this high mass fraction, MoS_2_ may act as a continuous matrix, rather than a coating on aggregated CNT bundles. This transition would activate additional deformation mechanisms to plastic deformation, such as CNT bundle pull-out from the matrix. Inspection of fracture surfaces at the low and high mass fraction end suggests this to be the case (Figure S13).

We further compared the stress transfer in the CNTF and the CNTF/MoS_2_ composite using cyclic tensile tests (Figure S14) conducted up to 1% strain rate, i.e., approximately in the linear region of the stress–strain plots mentioned above. After normalizing the results by the CNTF weight fraction, both the strength and modulus were comparable for the starting fabric and the composite. After the first cycle, there is a 0.3–0.4% nonrecoverable strain due to permanent interbundle stretching in the CNT network. In subsequent cycles, the modulus increases to 320 MPa for the pure fabric but only to 274 MPa for the composite. This can be taken as further indication of more realignment produced in the pure fabric during stretching, compared to the composite.

## 4 Conclusions

To summarize, we have demonstrated that DC heating is a rapid, targeted, and efficient technique for processing nanostructured composites of CNT bundle network and an inorganic matrix (MoS_2_). The maximum heating rate was as high as 31.7 °C/s. The processing method was well-controlled for all the studied mass fractions, which ranged from 54 to 68% MoS_2_, and corresponded to a coated bundle diameter between 123 and 295 nm, respectively. After Joule heating, the MoS_2_ transitioned into the crystalline 2H-phase while preserving the network structure of the composite and the primarily graphitic structure of the CNT network (D/G band intensity ratio ∼0.40). This opens a new processing route for similar CNT/inorganic composites for easy, out-of-oven manufacturing.

Joule-heated CNTF/MoS_2_ composites combine high longitudinal electrical conductivity [up to 1.72 (±0.25) × 10^5^ S/m] with high longitudinal tensile properties (specific modulus up to 8.82 ± 5.5 GPa) while preserving significant ductility (strain-to-break up to 3.7 ± 1.1%). When the tensile strength is normalized by density (up to 200 ± 58 MPa), Joule-heated CNTF/MoS_2_ composites are stronger than steel. The electronic conductivity of these composites is orders of magnitude higher than that obtained through wet-processing of nanostructured fillers, above the threshold for electron transport limitations in common battery electrodes, and approaching the level to eliminate metallic current collectors.

While the modulus can be described by a simple rule-of-mixture model, the composite strength can be described using a power law that varies with the CNTF mass fraction. This is attributed to the inorganic phase impeding the realignment and sliding of the CNT network upon stretching. More insights into the role of CNT alignment could be gained from in-situ orientation measurements, which will be the subject of a future study. Our composite system is also promising for metal-free battery anodes; the combination of high electrical conductivity and surface area of CNT bundles and the large interlayer spacing of MoS_2_ in these composites can potentially lead to applications in sodium-ion and dual-ion batteries. The exceptionally fast heating rates obtained through Joule heating (up to 31.7 °C/s) may enable thermal processing of other inorganic materials, such as metal oxides, for which it may be necessary to reduce annealing time to prevent oxidation of the conductive CNT network.

## Supporting Information Available

TGA, relationship of DC power density and electrical conductivity with MoS_2_ mass fraction, shifts in Raman peak position with Joule heating duration, XRD patterns, SEM images of the samples prior to Joule heating, distribution of CNT/MoS_2_ bundle diameter, calculation of MoS_2_ weight fraction as a function of MoS_2_ thickness, picture of the tensile testing setup, SEM images of the composite fracture surface, stress–strain curves before and after heating, and uniaxial cyclic tensile test results (PDF)
Thermal video (sped up 4×) and thermal profile of the Joule heating process (MP4)
