# Supplementary material for: Processing of Composite Electrodes of Carbon Nanotube Fabrics and Inorganic Matrices via Rapid Joule Heating
Source: ACS Appl Mater Interfaces. 2023 Jan 17;15(4):5590–9. doi: 10.1021/acsami.2c17901 (PMC10848196; doi:10.1021/acsami.2c17901)
Supplement: Supplementary file 1 — am2c17901_si_001.pdf [file am2c17901_si_001.pdf]

Supporting Information

Processing of composite electrodes of carbon nanotube fabrics and inorganic matrices via rapid Joule heating

Shegufta Upama[1,2], Anastasiia Mikhalchan[2], Luis Arévalo[2], Moumita Rana[3], Afshin Pendashteh[2], Micah J. Green[1,4*], Juan J. Vilatela[2*]

[1]Department of Materials Science & Engineering, Texas A&M University, College Station, TX 77843, USA.

[2]IMDEA Materials Institute, 28906 Getafe, Madrid, Spain.

[3]Institut für Anorganische und Analytische Chemie, University of Münster, Münster, Germany 48149.

[4]Artie McFerrin Department of Chemical Engineering, Texas A&M University, College Station, TX 77843, USA.

[*]Corresponding authors

Email address: juanjose.vilatela@imdea.org, micah.green@tamu.edu

A thermal video of the Joule heating process is included as a separate file (**Movie S1**).

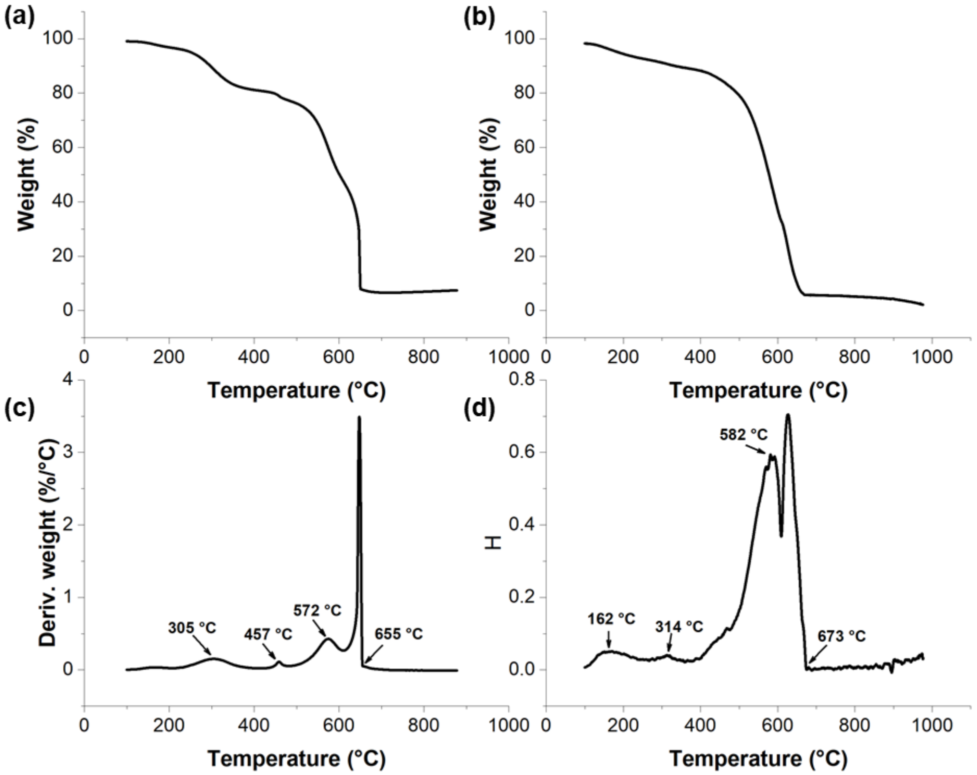

Figure S1: TGA curves for (a, c) pristine CNT fabric, and (b, d) functionalized CNT fabric.

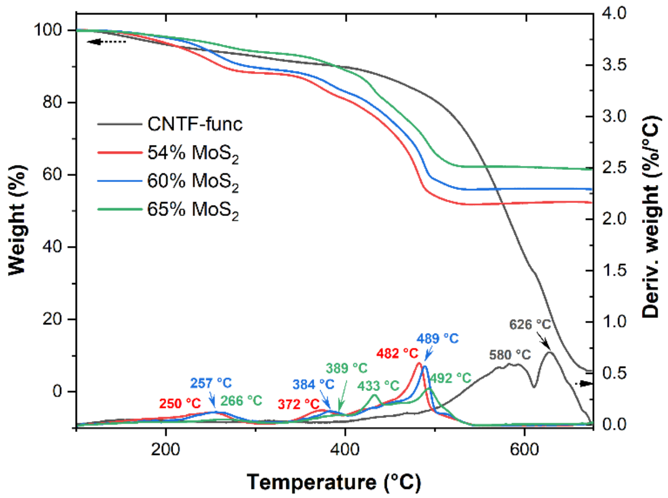

Figure S2: TGA curves for CNTF/MoS_2 composites with different weight fractions compared to the functionalized CNT fabric.

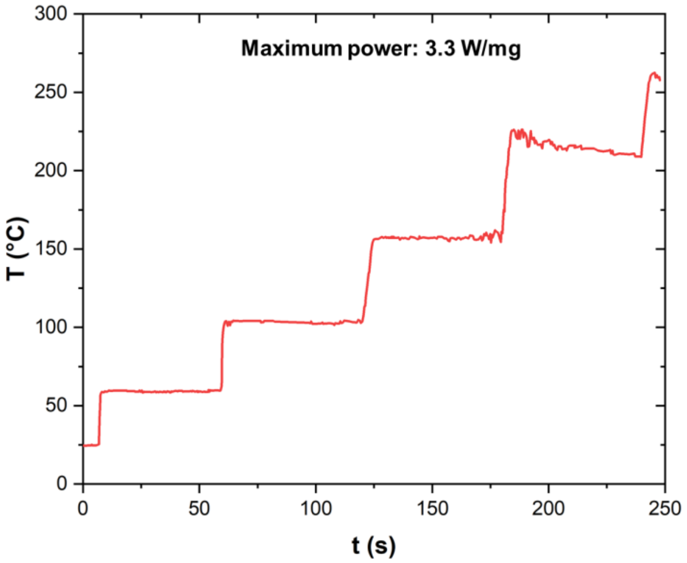

Figure S3: Temperature profile with time for the Joule heating video (**Movie S1**). Note that the maximum temperature was ~250 °C due to the calibration limit of the FLIR camera.

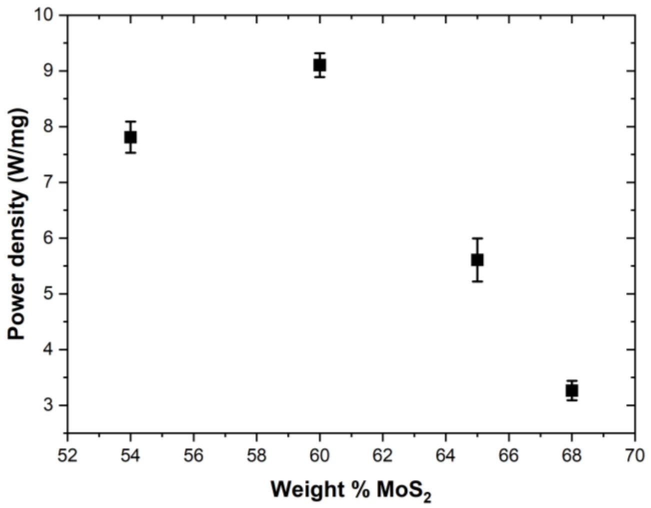

Figure S4: Relationship of power density with the composite mass fraction. Here, power refers to the maximum power at the target temperature, which was normalized by the sample mass.

Table S1: Raman peak positions and intensity ratios of CNTF/MoS_2 (54% MoS_2) for different DC-heating durations

Annealing time	Raman shift				D/G ratio	A_{1g}/G ratio
	E^1_{2g}	A_{1g}	D	G		
0 min	-	-	1348.81 ± 0.61	1580.60 ± 0.51	0.46 ± 0.01	-
5 min	381.66 ± 0.14	408.58 ± 0.07	1352.31 ± 0.39	1586.39 ± 0.20	0.46 ± 0.01	10.19 ± 0.39
10 min	382.24 ± 0.06	407.76 ± 0.05	1348.88 ± 0.29	1585.31 ± 0.20	0.38 ± 0.00	5.33 ± 0.18
15 min	382.01 ± 0.08	408.75 ± 0.05	1354.53 ± 0.35	1584.33 ± 0.17	0.43 ± 0.01	5.24 ± 0.18

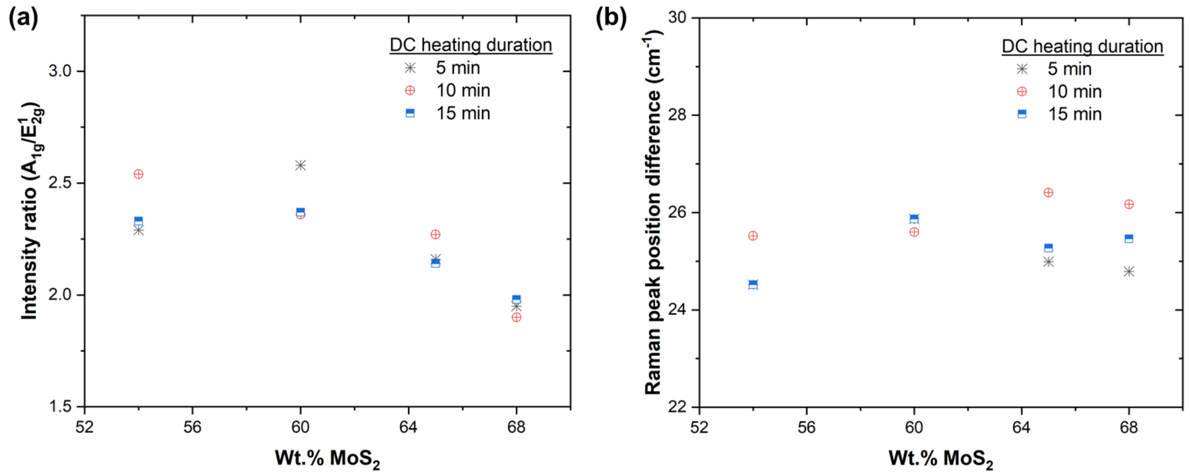

Figure S5: (a) Intensity ratio of A_{1g}/E^1_{2g} Raman peaks, and (b) frequency difference between the two peaks with respect to the DC heating duration and mass fraction of the composite.

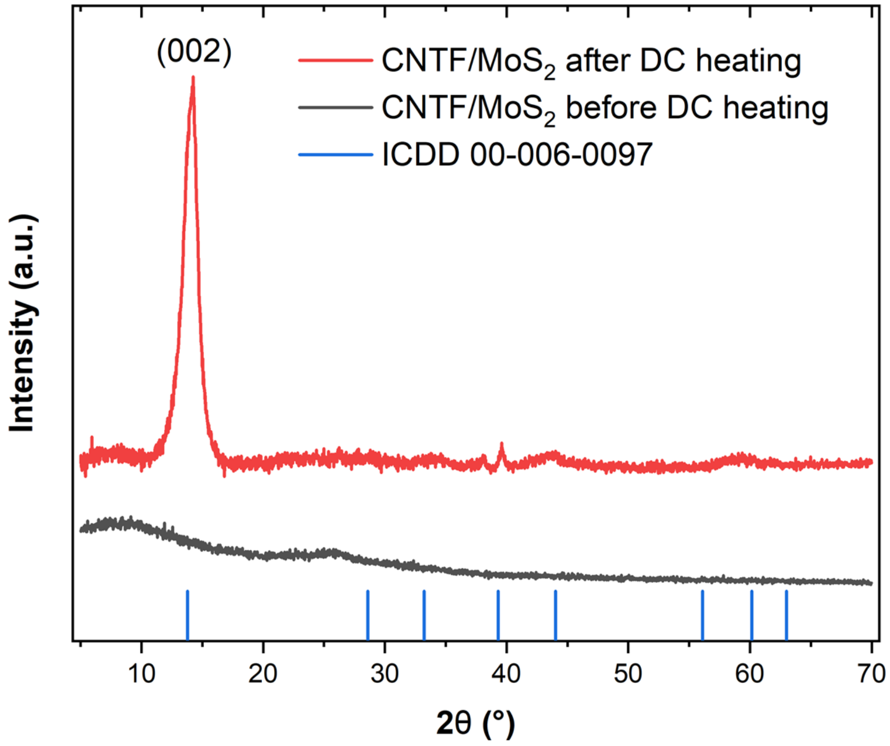

Figure S6: XRD pattern for CNTF/MoS_2 (60% MoS_2), before and after DC heating. The DC-heated composite shows the predominant (002) basal plane. The blue reference peaks correspond to 2H-phase MoS_2.

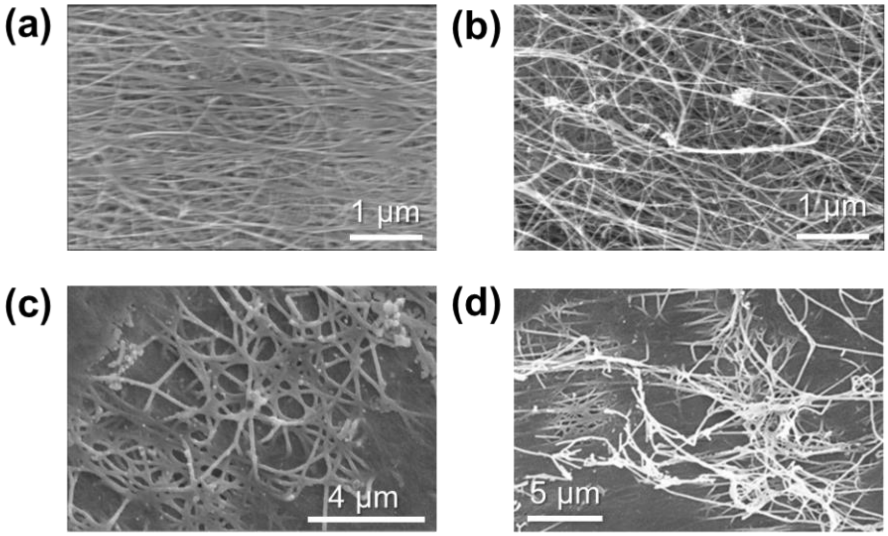

Figure S7: FE-SEM images of CNT fabric and CNTF/MoS_2 composites prior to DC heating. (a) As-synthesized (pristine) CNT fabric, (b) functionalized (ozone-treated) CNT fabric, (c) 54% MoS_2 composite, and (d) 68% MoS_2 composite.

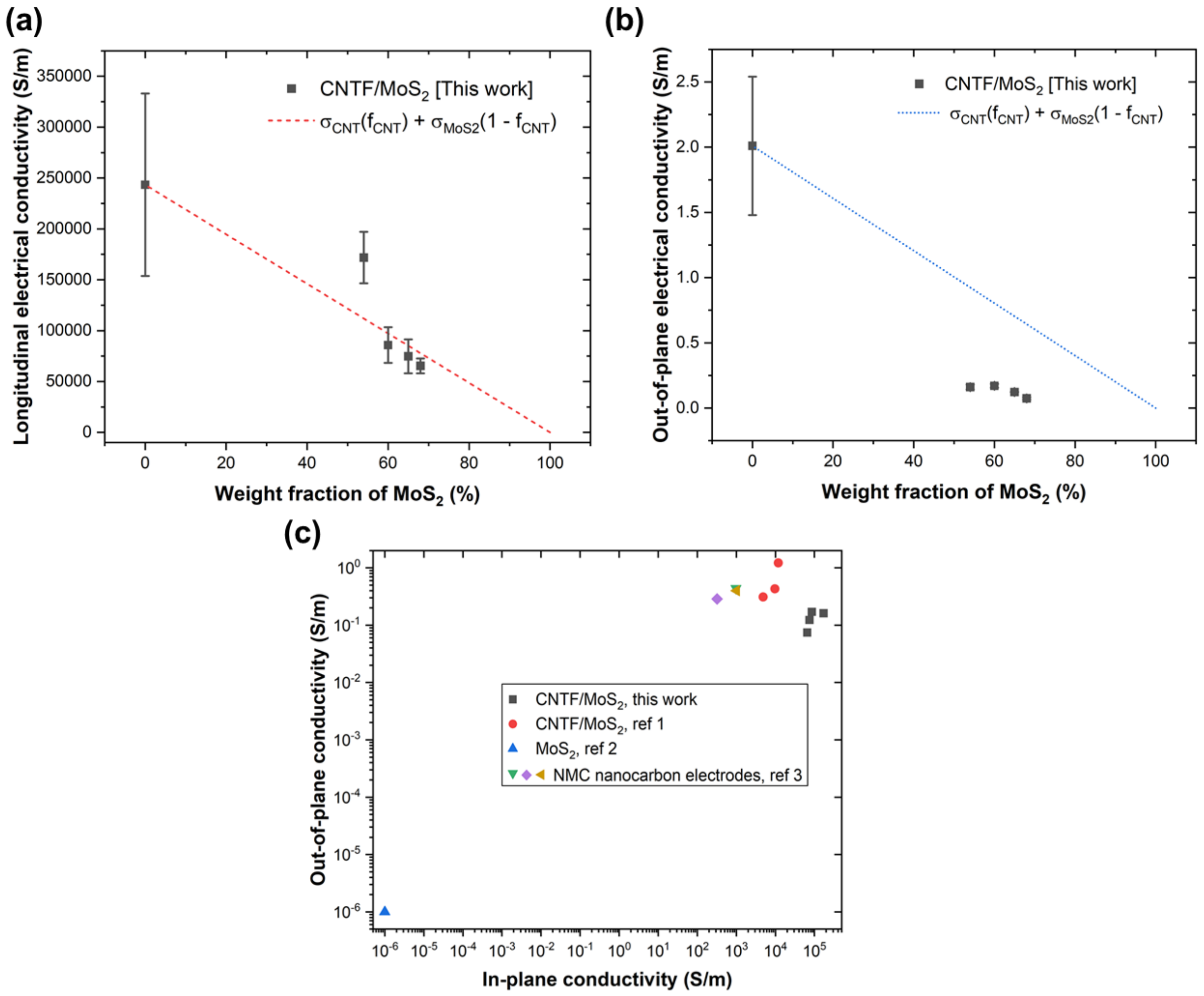

Figure S8: Relationship of (a) longitudinal electrical conductivity and (b) out-of-plane electrical conductivity as a function of the composite mass fraction. The dotted lines compare electrical conductivity of the CNTF/MoS_2 composite with the rule-of-mixtures model. (c) Comparison of electrical conductivity in this work with prior work on CNTF/MoS2, pure MoS2, and other nanocomposites.[1], [2], [3]

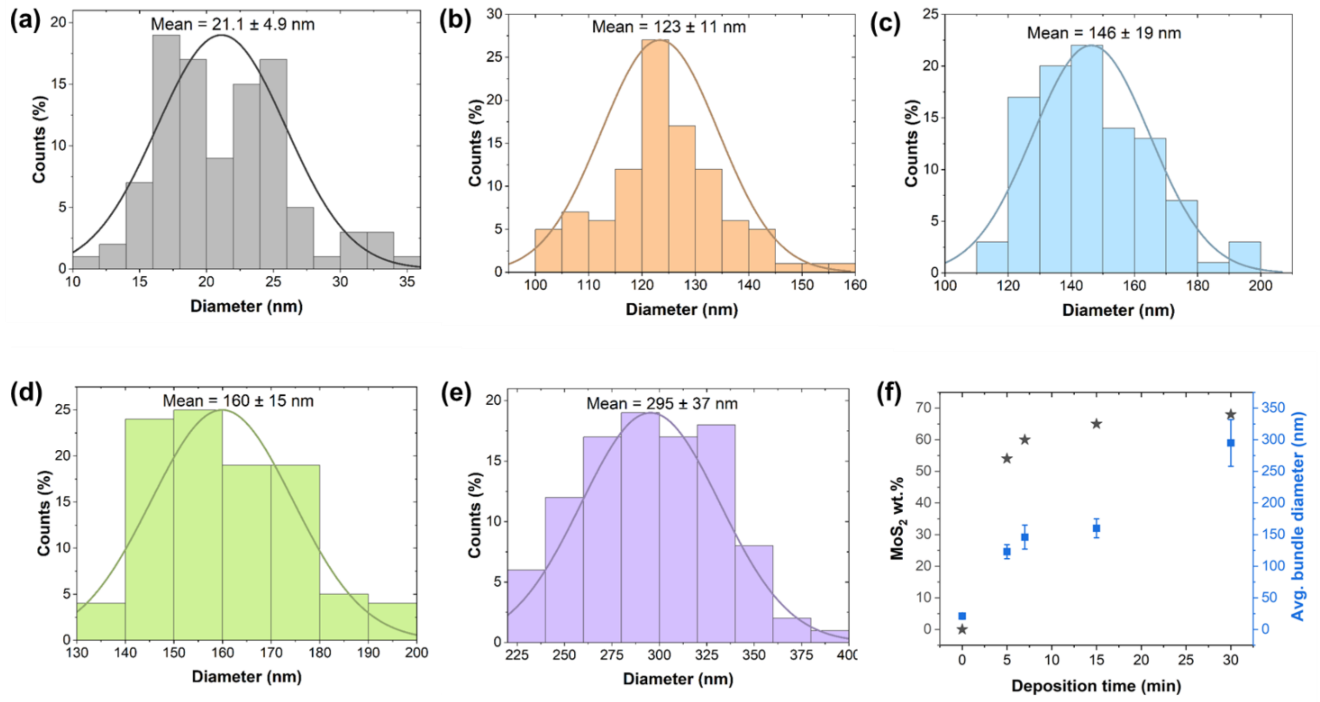

Figure S9: Distribution of (a) CNT bundle core diameter, and (b-e) CNT/MoS_2 bundle diameter. (f) Dependence of the bundle diameter and mass fraction of the composite on MoS_2 deposition time.

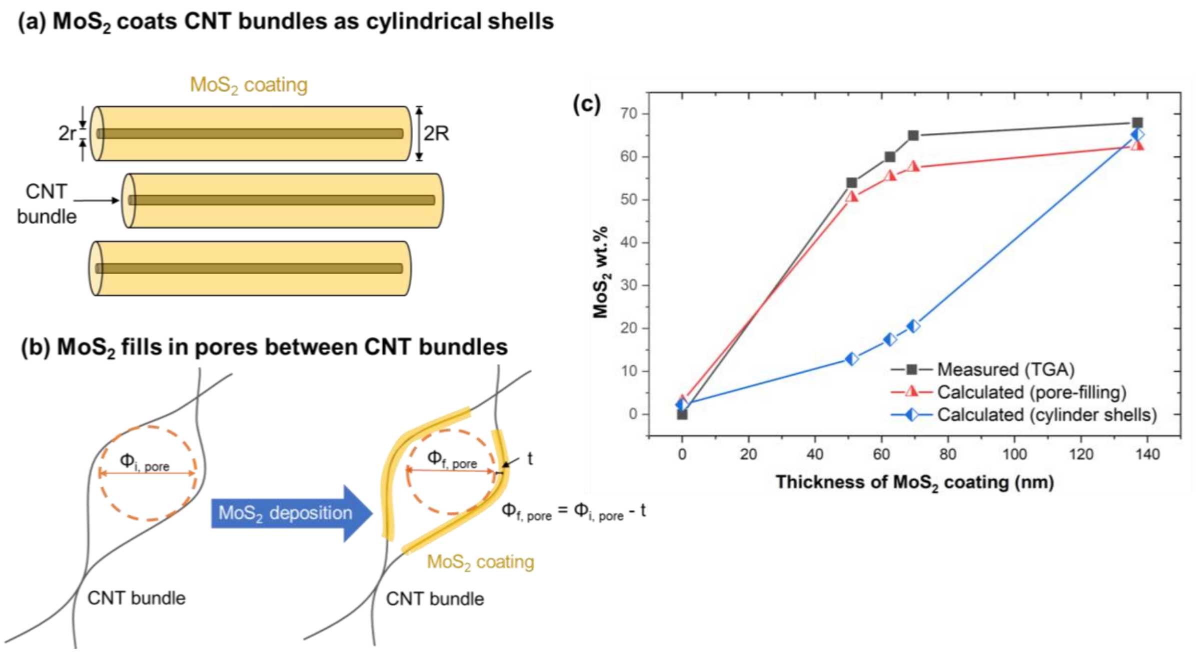

Figure S10: Schematic of MoS_2 coating as (a) cylindrical shells around CNT bundles, and (b) filling in pores between the CNT bundles. (c) The dependence of MoS_2 weight fraction on the MoS_2 coating thickness resembles a pore-filling model rather than a cylindrical shell model.

Calculations:

(i) Assuming that the MoS_2 coating is a cylindrical shell around each CNT bundle, the mass fraction of MoS_2 is:

$$m_f \propto \frac{A_{MoS2}}{A_{MoS2}+A_{CNT}} = \frac{R^2-r^2}{R^2}, \text{ (Eq. S1)}$$

where, A_{MoS2} = Area of MoS_2 shell, A_{CNT} = Area of CNT bundle, R = radius of MoS2 shell, and r = radius of CNT bundle.

(ii) Assuming that the MoS_2 fills in spherical pores formed by overlapping CNT bundles:

Mass fraction of MoS_2, $m_f \propto Initial\ porosity - Final\ porosity$

$$m_f \propto (\Phi_{i,pore})^3 - (\Phi_{i,pore} - t)^3, \text{ (Eq. S2)}$$

where, $\Phi_{i,pore}$ = Initial diameter of pore, prior to deposition of MoS_2, t = Thickness of the MoS_2 coating around CNT bundle (see **Table 1**), and $(\Phi_{i,pore} - t)$ = Final diameter of pore, after MoS_2 deposition

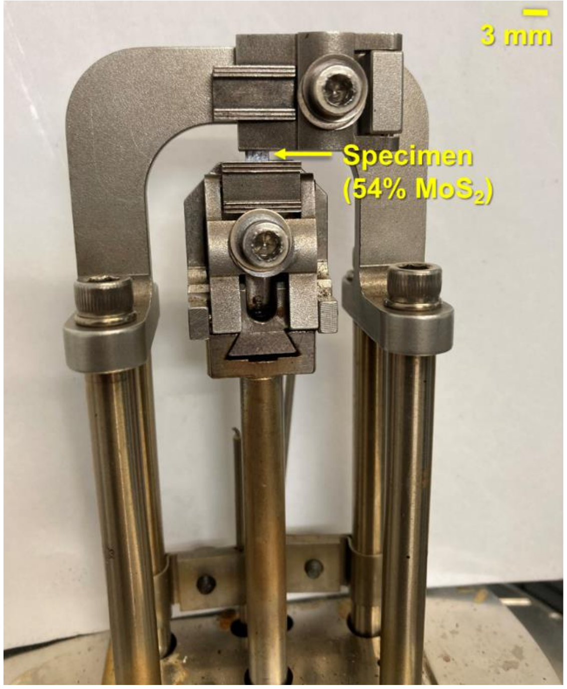

Figure S11: Tensile testing setup in a Dynamic Mechanical Analyzer (DMA)

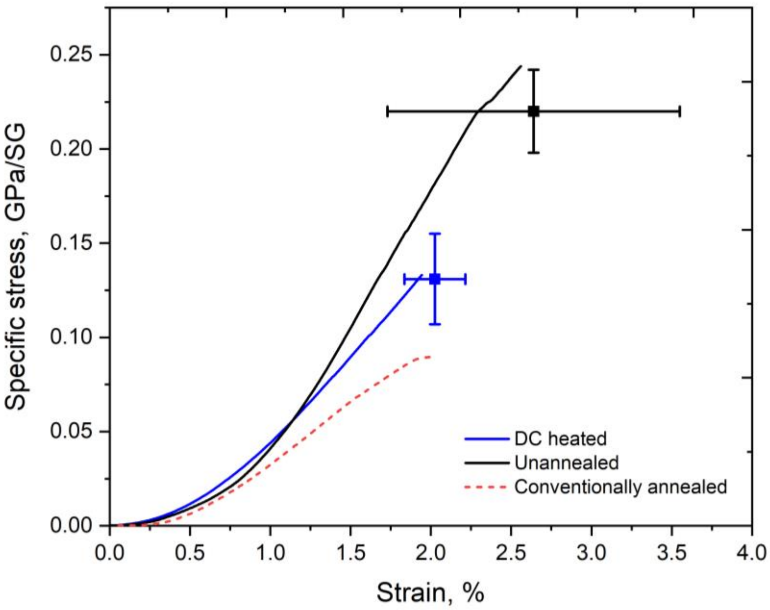

Figure S12: Stress-strain curves for the 54% MoS_2 composite before and after annealing, using either a tube furnace or DC heating

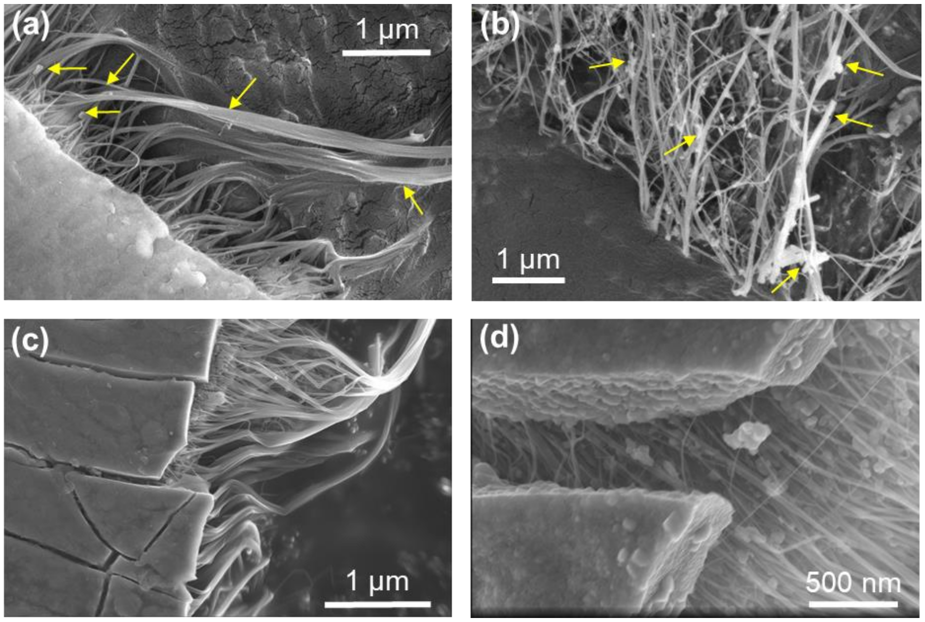

Figure S13: SEM images of the CNTF/MoS_2 composite fracture surface for (a, b) a relatively low weight fraction of 54% MoS_2, and (c, d) a relatively high weight fraction of 68% MoS_2. The 54% composite has residual MoS_2 coating (opaque, shown using yellow arrows) on the CNT bundles after fracture. In contrast, the CNT bundles (transparent) pull out from the MoS_2, which fractures as a continuous matrix.

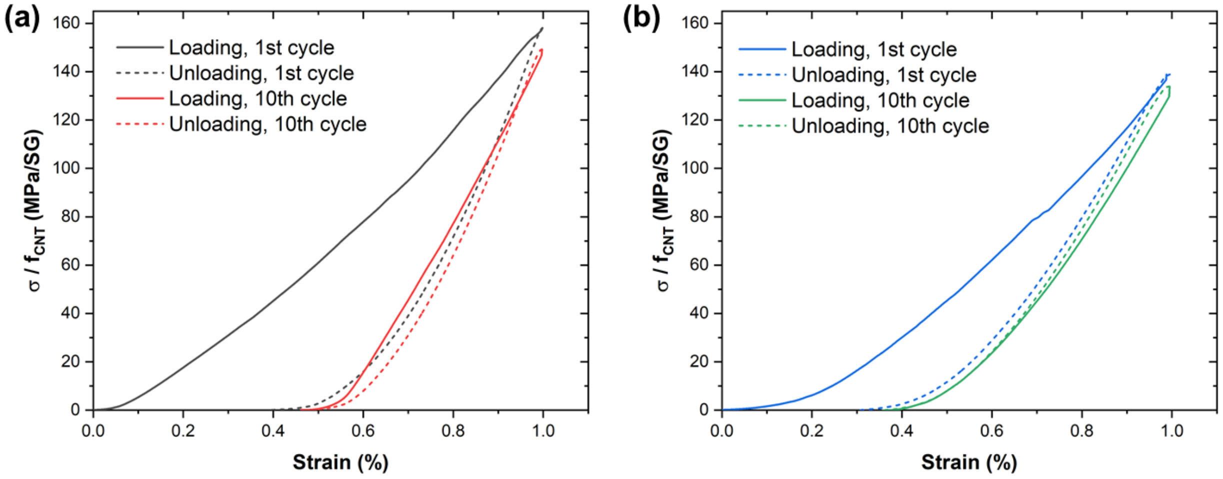

Figure S14: Uniaxial cyclic tensile test results for (a) a functionalized CNT fabric specimen, and (b) a 60% MoS_2 composite specimen. The specific stress (σ) was normalized by the weight fraction of the CNT fabric (f_{CNT}).

References

(1) Rana, M.; Boaretto, N.; Mikhalchan, A.; Vila Santos, M.; Marcilla, R.; Vilatela, J. J. Composite Fabrics of Conformal MoS2 Grown on CNT Fibers: Tough Battery Anodes without Metals or Binders. *ACS Appl. Energy Mater.* **2021**. https://doi.org/10.1021/acsaem.1c00482.

(2) El-Mahalawy, S. H.; Evans, B. L. Temperature Dependence of the Electrical Conductivity and Hall Coefficient in 2H-MoS2, MoSe2, WSe2, and MoTe2. *Phys. Status Solidi B* **1977**, *79* (2), 713–722. https://doi.org/10.1002/pssb.2220790238.

(3) Tian, R.; Alcala, N.; O'Neill, S. J. K.; Horvath, D. V.; Coelho, J.; Griffin, A. J.; Zhang, Y.; Nicolosi, V.; O'Dwyer, C.; Coleman, J. N. Quantifying the Effect of Electronic Conductivity on the Rate Performance of Nanocomposite Battery Electrodes. *ACS Appl. Energy Mater.* **2020**, *3* (3), 2966–2974. https://doi.org/10.1021/acsaem.0c00034.
